# A Scoping Review of Social Housing Models for Older Adults

**DOI:** 10.1093/geroni/igaa057.3346

**Published:** 2020-12-16

**Authors:** Christine Sheppard, Matthew Yau, Carol Kwon, Jorge Rios, Andrea Austen, Sander Hitzig

**Affiliations:** 1 Sunnybrook Research Institute, Toronto, Ontario, Canada; 2 University of Toronto, University of Toronto, Ontario, Canada; 3 University of Toronto, Toronto, Ontario, Canada; 4 City of Toronto, Toronto, Ontario, Canada; 5 Sunnybrook Hospital, North York, Ontario, Canada

## Abstract

Access to affordable housing is a rising concern for many older adults, and government-sponsored social housing programs are one approach to support low-income older adult renters; however, these housing models are limited in availability and may not all be well-suited to support aging in place. To better understand how to promote the physical, mental and social wellbeing of older tenants in social housing, this scoping review mapped relevant literature to examine: 1) the characteristics of older adults in social housing; and 2) social housing service models and policies. Seven peer reviewed databases were searched for relevant articles, which were screened by two independent reviewers. A total of 140 articles met the inclusion criteria. Studies were predominately from the US and Canada; spanning over five decades of research, with publications surging in the 1980’s and in the 2010’s. Almost all studies reported on the sociodemographic and health characteristics of older tenants, and two thirds presented findings on social housing service models, including policies, staff positions and training, and access to on-site support services. This review points to a high level of vulnerability among older adult tenants living in social housing and highlights the importance of co-locating support services in social housing buildings, with dedicated tenant-support staff to identify vulnerable tenants and link them to these services. There is an acute need for more research on key issues related to housing retention, such as eviction prevention, in order to identify opportunities for social housing landlords to help older tenants age in place.

